# A paradigm shift toward full-cycle management of atrial fibrillation: integrating digital twins and artificial intelligence

**DOI:** 10.3389/fcvm.2026.1872233

**Published:** 2026-06-22

**Authors:** Dandan Song, Shaning Yang

**Affiliations:** Department of Cardiology, The First Affiliated Hospital of Yangtze University, Jingzhou, China

**Keywords:** artificial intelligence, atrial fibrillation, digital twin, full-cycle management, personalized medicine, virtual closed-loop management

## Abstract

**Background:**

Clinical management of atrial fibrillation (AF) often remains fragmented, with decisions largely based on information available at a single time point rather than on the continuous evolution of the disease. Emerging technologies such as digital twins and artificial intelligence (AI) offer new possibilities to change this paradigm by enabling dynamic, patient specific modelling and data driven decision support across the full disease cycle.

**Objective:**

This review comprehensively examines the technical pathways and clinical value of integrating digital twins and artificial intelligence across the full cycle of atrial fibrillation, and explores the potential impact of this integration on diagnostic and treatment paradigms. In the present review, the concept of virtual closed-loop AF management encompasses both: (1) real-time or near-real-time decision support during in-hospital electrophysiological procedures; (2) longitudinal post-recording analysis based on wearable devices, ambulatory ECG monitoring, imaging datasets, and electronic health records. Therefore, the proposed framework spans outpatient screening, in-hospital intervention planning, intra-procedural guidance, and post-procedural follow-up management.

**Methods:**

A structured narrative literature review was conducted to summarize recent advances in digital twin technology and artificial intelligence for atrial fibrillation management. Particular attention was given to multimodal data integration, patient-specific modeling, in silico simulation, and AI-assisted clinical decision support.

**Results:**

A relatively complete technical chain for constructing patient-specific cardiac digital twins has been established, achieving anatomical Dice coefficients of 93% or higher and a correlation coefficient for activation time prediction exceeding 0.96. Artificial intelligence permeates the entire process of AF management: the use of AI-ECG increased AF detection rates by 2.3-fold, models for predicting post-ablation recurrence achieved area under the curve (AUC) values generally ranging from 0.72 to 0.85, and intra-operative three-dimensional reconstruction time was reduced to 65 s. The integration of the virtual closed-loop framework: “data sensing → model construction → in silico testing → clinical decision → feedback optimisation” has been preliminarily validated in scenarios such as drug screening, ablation planning, and thrombus risk assessment.

**Conclusion:**

The integration of digital twin and artificial intelligence provides a novel pathway for full-cycle atrial fibrillation management, and holds the potential to shift the current diagnostic and therapeutic paradigm from fragmentation towards continuity. The first prospective multicentre randomised controlled trial has provided the first prospective evidence supporting Al-assisted personalized AF management; however, its clinical application still relies on further validation through multicentre studies and high-quality evidence. As technology matures and evidence accumulates, this model is expected to be gradually introduced into clinical practice.

## 1 Introduction: clinical challenges in atrial fibrillation management and the need for a new paradigm

Atrial fibrillation is one of the most common cardiac arrhythmias worldwide, and its management trajectory is often fragmented, encompassing multiple steps such as screening, stroke risk assessment, rhythm control, and anticoagulation therapy. However, in real-world clinical practice, patients often receive care across different healthcare settings, and decisions at each stage are largely based on partial information available at a single time point, lacking dynamic assessment and integration of the entire disease course.

Although the CHA₂DS₂-VASc score can be used for stroke risk stratification, it struggles to predict when an individual patient will experience a stroke; pre-procedural imaging can provide anatomical guidance for ablation planning, but fails to identify the true arrhythmogenic substrate; post-procedural imaging and procedural follow-up relies mainly on periodic ECG monitoring or incidental symptom recording, leaving patients with asymptomatic recurrences easily missed. The essence of the above challenges lies in the lack of a unified platform for AF management that can integrate multi-source data, dynamically predict disease progression, and guide treatment decisions (12, 13).

In recent years, the rapid development of digital twins and artificial intelligence technologies has opened up new possibilities for addressing the above challenges. Digital twins integrate multiple-source data such as imaging, electrophysiology, and wearable devices to construct a virtual mirror model of the patient, enabling dynamic prediction of cardiac structure and electrical activity (8, 14). Artificial intelligence, in turn, can process massive amounts of high-dimensional data, identify complex nonlinear patterns, and optimise predictive models, thereby supporting risk assessment and treatment decision-making.

In recent years, particularly since 2024–2025, an increasing number of reviews have focused on digital twin and artificial intelligence applications in atrial fibrillation. Currently, relevant reviews have mostly focused either on the construction of digital twin models or on the application of artificial intelligence algorithms in atrial fibrillation (15, 16); there is a lack of studies that comprehensively integrate the two from a clinical pathway perspective. An overarching framework of “data acquisition – model construction – decision support – feedback optimisation” has not yet been established.

Unlike previous technology-centered reviews that mainly focused on isolated applications of digital twin construction or artificial intelligence algorithms, the present review emphasizes a clinically integrated virtual closed-loop framework linking multimodal data acquisition, patient-specific modeling, in silico simulation, treatment planning, and iterative clinical feedback.

The main innovation of this article lies in the following aspects: (1) starting from the clinical management workflow, we propose a full-cycle virtual closed-loop management framework for atrial fibrillation comprising “data sensing – model construction – in silico testing – clinical decision – feedback optimisation”; (2) we construct a technology readiness–evidence strength matrix to provide a structured evaluation of the level of clinical translation of technologies related to digital twins and artificial intelligence; (3) based on current evidence, we analyse the prospects for its application in clinical practice and propose key research directions for the next 3-5 years. Collectively, this work aims to provide a reference for transforming AF management from the traditional fragmented model towards a continuous, individualised paradigm. In the following sections, we describe how a patient's cardiac anatomy and electrophysiology can be replicated as a digital twin, enabling in silico analysis and treatment (e.g., ablation) personalization.

## 2 Literature search strategy

A structured narrative literature review was conducted to summarize recent advances in digital twin technology and artificial intelligence for atrial fibrillation management. PubMed, Web of Science, Embase, and Google Scholar databases were searched for studies published up to February 2026. Search terms included combinations of “atrial fibrillation”, “digital twin”, “artificial intelligence”, “machine learning”, “computational modeling”, “electrophysiological modeling”, “ablation planning”, “risk prediction”, and “in silico simulation”. The initial search identified approximately 81 records. After removal of duplicates and screening for relevance, 43 studies were considered eligible and included in the present narrative review.

Priority was given to representative original studies, clinically relevant translational investigations, multicenter studies, and influential methodological reports. Conference abstracts without full datasets, non-English publications, and purely experimental studies without translational implications were excluded. The present review was designed as a clinically oriented narrative synthesis rather than a formal systematic review or meta-analysis.

## 3 Methods for constructing patient-specific cardiac digital twins

A digital twin differs from conventional computational models in that it continuously incorporates patient-specific data and can be iteratively updated to reflect the evolving clinical state of the individual. A digital twin serves as the foundation for achieving virtual closed-loop management, with its core lying in constructing a dynamic cardiac model that accurately reflects an individual's cardiac anatomy and electrophysiological properties. In the digital twin paradigm, patient-specific anatomical, electrophysiological, imaging, and clinical data are integrated to generate a virtual representation of the individual patient. This digital counterpart can then be used to simulate disease progression, evaluate potential treatment strategies, and perform in silico testing of catheter ablation scenarios before clinical implementation. The resulting predictions may support clinical decision-making while reducing the need for empirical trial-and-error approaches. Although multimodal imaging plays a central role in anatomical reconstruction and fibrosis characterization, electrophysiological information derived from surface ECGs, intracardiac electrograms, and electroanatomical mapping systems remains essential for identifying arrhythmogenic substrates and guiding ablation strategies.

### 3.1 Structural and pathological modeling based on multimodal imaging

The first step in constructing a digital twin is to obtain an accurate three-dimensional anatomical structure of the atria. In recent years, the application of deep learning has markedly improved the automation and accuracy of image segmentation. The study by Buongiorno et al. developed a 3D residual U-Net algorithm capable of performing time-resolved segmentation of ECG-gated CT images, enabling automatic quantification of left atrial volume and providing an efficient tool for dynamic monitoring of atrial remodelling (17). Myocardial fibrosis, as a key arrhythmogenic substrate for AF, can also be segmented simultaneously using deep learning. Yang et al. proposed the multi-view transformer with tensor attention (MVTT), which integrates multi-view information and an attention mechanism to simultaneously and automatically segment left atrial anatomy and scar tissue. In independent testing, the model achieved a Dice coefficient of 93.11% for anatomical segmentation and 86.59% for scar segmentation, with a processing time of approximately 0.27 s per case (1). The quality of training data is key to determining model performance. Studies have shown that optimising the specific imaging protocol for late gadolinium enhancement magnetic resonance imaging (LGE-MRI) can effectively improve the accuracy of AI-based segmentation (18). Beyond macroscopic imaging, Kulathilaka et al. incorporated micrometre-resolution imaging data into digital twin construction, enabling the visualisation of microstructural features such as myocardial fibre organisation and fatty infiltration. This provides a new structural perspective for understanding the mechanisms underlying AF maintenance (19).

### 3.2 Personalised calibration of electrophysiological properties based on inverse problems

On the basis of the anatomical model, it is necessary to further assign electrophysiological properties to it. Inverse modeling approaches can use limited clinical electrophysiological data to infer personalised conduction properties. Lubrecht et al. developed the PIEMAP method, which uses sparse intracardiac contact mapping recordings to inversely estimate local fibre orientation and anisotropic conduction velocity in the left atrium. The predicted activation times in unobserved regions correlated highly with actual measurements (*r* ≈ 0.96). The estimated conduction velocity along the fibre direction was approximately 82 ± 25 cm/s, and perpendicular to the fibre direction it was approximately 46 ± 7 cm/s, with an anisotropic ratio of about 1.91 ± 0.16 (2). The study by Abdi et al. used intracardiac electrograms to estimate tissue conductivity (20), and Zappon et al. developed an end-to-end framework to invert body surface ECG signals into parameters for three-dimensional volumetric models (21). Díaz et al. found that personalising parameters such as the effective refractory period substantially influences the assessment of arrhythmia vulnerability in computer models, thereby highlighting the need for precise parameter calibration (22).

### 3.3 Clinical validation of model phenotypes

The constructed digital twin then needs to undergo rigorous clinical validation. Macheret et al. built personalised left atrial models of persistent AF patients using LGE-MRI (23). By adjusting conduction velocity, they improved the agreement between simulated arrhythmia induction and clinical recurrence phenotypes (AF, atrial flutter (AFL), or no recurrence) from 46% to 65%, suggesting that personalising model parameters may help improve predictive accuracy. Computer simulation studies have also shown that electrophysiological parameters such as P-wave duration are associated with the risk of post-ablation recurrence, and the underlying mechanism may be related to increased atrial conduction velocity and a shortened effective refractory period (24). Collectively, these studies indicate that a well-calibrated digital twin can accurately reflect the patient's clinical phenotype, thereby laying the foundation for subsequent in silico testing and decision support.

## 4 Artificial intelligence: the core of perception and decision-making driving the closed loop

Artificial intelligence is a key technology that drives the operation of virtual closed-loop management, spanning multiple stages including data acquisition, risk prediction, and clinical decision-making.

### 4.1 Early warning and risk prediction

AI-enabled ECG analysis has become an important tool for early screening of AF. Noseworthy et al. conducted a prospective non-randomised interventional trial showing that applying an AI algorithm to sinus-rhythm ECGs could identify individuals at high risk of AF, increasing AF detection rates by 2.3-fold (3). Randomised clinical trials further confirmed that AI-ECG not only improves AF detection but also promotes appropriate use of anticoagulation therapy (25). For predicting post-ablation recurrence, various machine learning approaches have shown good performance. Saglietto et al.developed the AFA-Recur online calculator based on data from a European AF registry, achieving an AUC of 0.72 for predicting recurrence (4). Combining clinical and imaging features can further improve predictive performance (5). Moreover, Asaeikheybari et al. performed a morphological analysis of the pulmonary veins using CT imaging, which was also found to be associated with the risk of recurrence (6). Although these AI-enabled risk prediction models have demonstrated promising predictive performance, current evidence is primarily derived from retrospective cohorts and relatively limited sample sizes.

### 4.2 Intra-procedural real-time guidance and ablation target identification

Catheter ablation is a rhythm-control procedure in which targeted thermal energy is delivered to arrhythmogenic atrial tissue in order to electrically isolate pulmonary veins or interrupt abnormal conduction pathways. In current electrophysiological practice, intracardiac mapping catheters are used to record local electrical activity prior to ablation in order to identify abnormal conduction regions. The study by Di Biase et al. demonstrated that a deep learning-based intracardiac echocardiography reconstruction technique could generate a three-dimensional model of the left atrium in approximately 65 s, which correlated well with CT findings, and achieved a 100% acute success rate in 28 patients undergoing AF ablation (7). Machine learning algorithms have also been used to identify pulmonary vein isolation status, thereby enabling real-time intra-procedural assessment (26). For identifying ablation targets, Liu et al. used deep learning to predict the origin of trigger sites in patients with paroxysmal AF (27), and Rodrigo et al. applied deep learning to recognise characteristic signals of AF on intracardiac electrograms, thereby providing a powerful tool for precise ablation (28).

### 4.3 Post-procedural management and prognostic evaluation

In the post-procedural period, AI can integrate multi-source signals to improve prognostic assessment. For example, Tang et al. found that a model fusing intracardiac and body surface signals significantly improved the accuracy of recurrence prediction (29). Similarly, Shade et al. found that combining mechanistic models with machine learning could also predict the risk of post-ablation recurrence before the procedure (30). For patients with cardiac implantable electronic devices, Kim et al. developed an AI algorithm capable of effectively identifying clinically relevant high-rate atrial episodes (31). Collectively, these studies have demonstrated that AI has already permeated the entire process of AF management, serving as an important foundation for building a closed-loop system.

## 5 Integration framework and clinical translation of the virtual closed-loop management framework

In clinical practice, digital twins do not replace treatment performed on the real patient. Instead, multimodal imaging and electrophysiological datasets are first used to construct a patient-specific computational replica of the atria. Different intervention strategies can subsequently be tested in silico on the virtual model before the actual procedure is performed. This approach may help optimize lesion placement, improve procedural efficiency, and reduce unnecessary ablation.

### 5.1 System architecture and workflow

**Figure 1:**
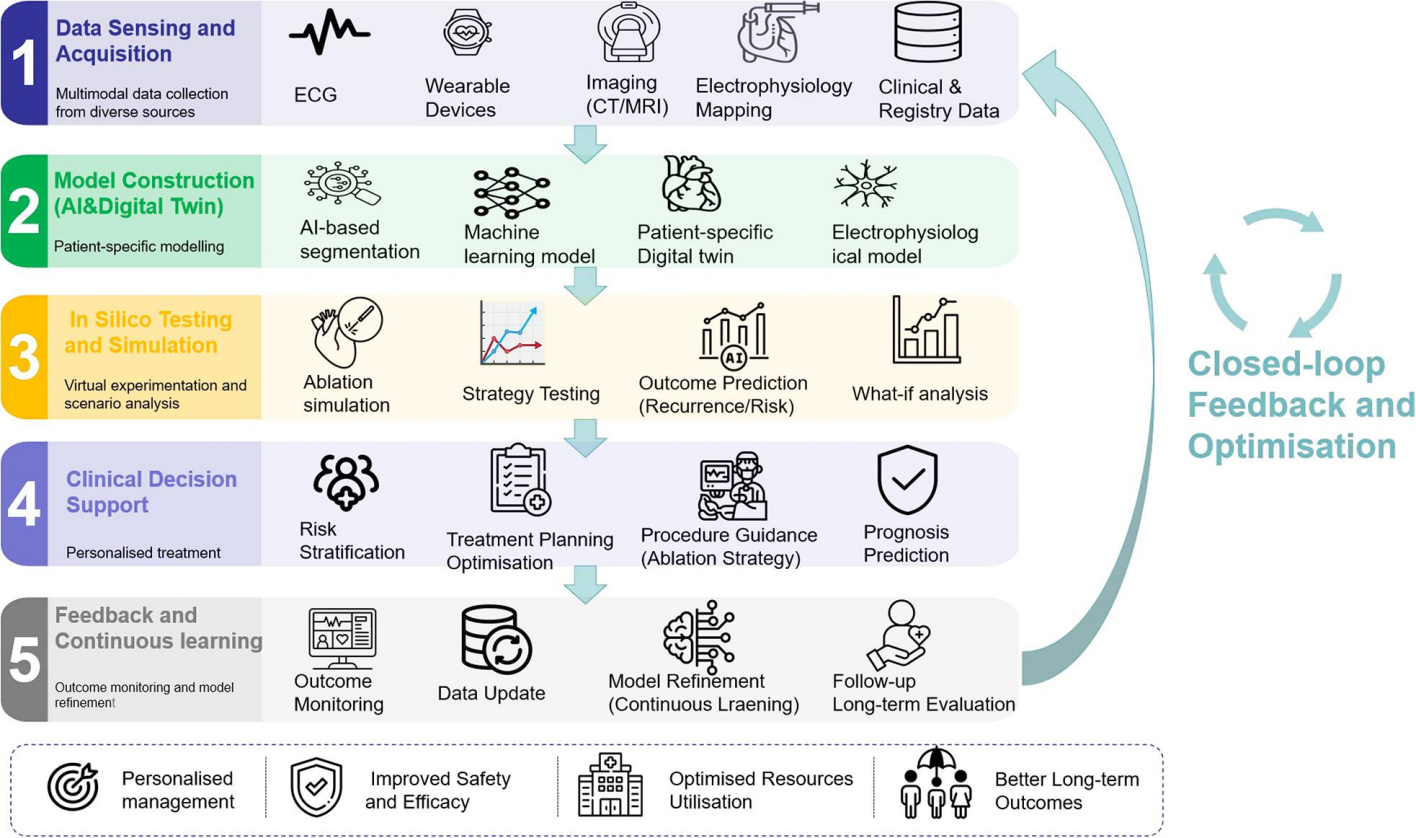
Virtual closed-loop framework for full-cycle management of atrial fibrillation integrating digital twin and artificial intelligence. The framework consists of five layers that correspond to Sections 5.1 ①-⑤:①Data Sensing (integrating ECG, wearables, imaging, electrophysiological mapping, and clinical data), ②Model Construction (AI-based segmentation, machine learning, and patient-specific digital twin with electrophysiological modelling),③In Silico Testing (ablation simulation, strategy testing, outcome prediction, and what-if analysis),④Clinical Decision Support (risk stratification, treatment planning, and prognosis prediction), and ⑤Feedback & Continuous Learning (outcome monitoring, data update, model refinement, and long-term evaluation). Arrows indicate the iterative closed-loop flow. The bottom boxes summarise expected clinical benefits (personalised management, improved safety/efficacy, optimised resources, better long-term outcomes).

Building on the above technologies, a virtual closed-loop management framework covering the full cycle of AF can be constructed. The architecture of the proposed virtual closed-loop system is illustrated in Figure 1, the core workflow of the virtual closed-loop system consists of five interconnected layers: ① Data sensing layer (left side of Figure 1): This layer integrates multi-source information such as wearable device ECG signals (3, 25), imaging data(CT/MRI) (1, 17), intra-procedural electrophysiological recordings (2); and clinical data (e.g., electronic health records) (32); ② model construction layer (second column of Figure 1): This layer generates patient-specific anatomical and electrophysiological models using deep learning algorithms (1, 21), and calibrating key parameters through inverse methods (2, 22); ③ in silico testing and simulation layer (middle of Figure 1): Here, different intervention strategies are simulated on the digital twin, including ablation line configuration (9), response to antiarrhythmic drugs (8), or the effect of left atrial appendage closure (33); ④ clinical decision support layer(fourth column of Figure 1): Simulation results are combined with predictive models (4, 5) to provide clinicians with visual decision support;⑤feedback optimisation and continuous learning layer(right side of Figure 1): real-world treatment outcomes are fed back into the model to enable continuous iteration.

**Figure 2:**
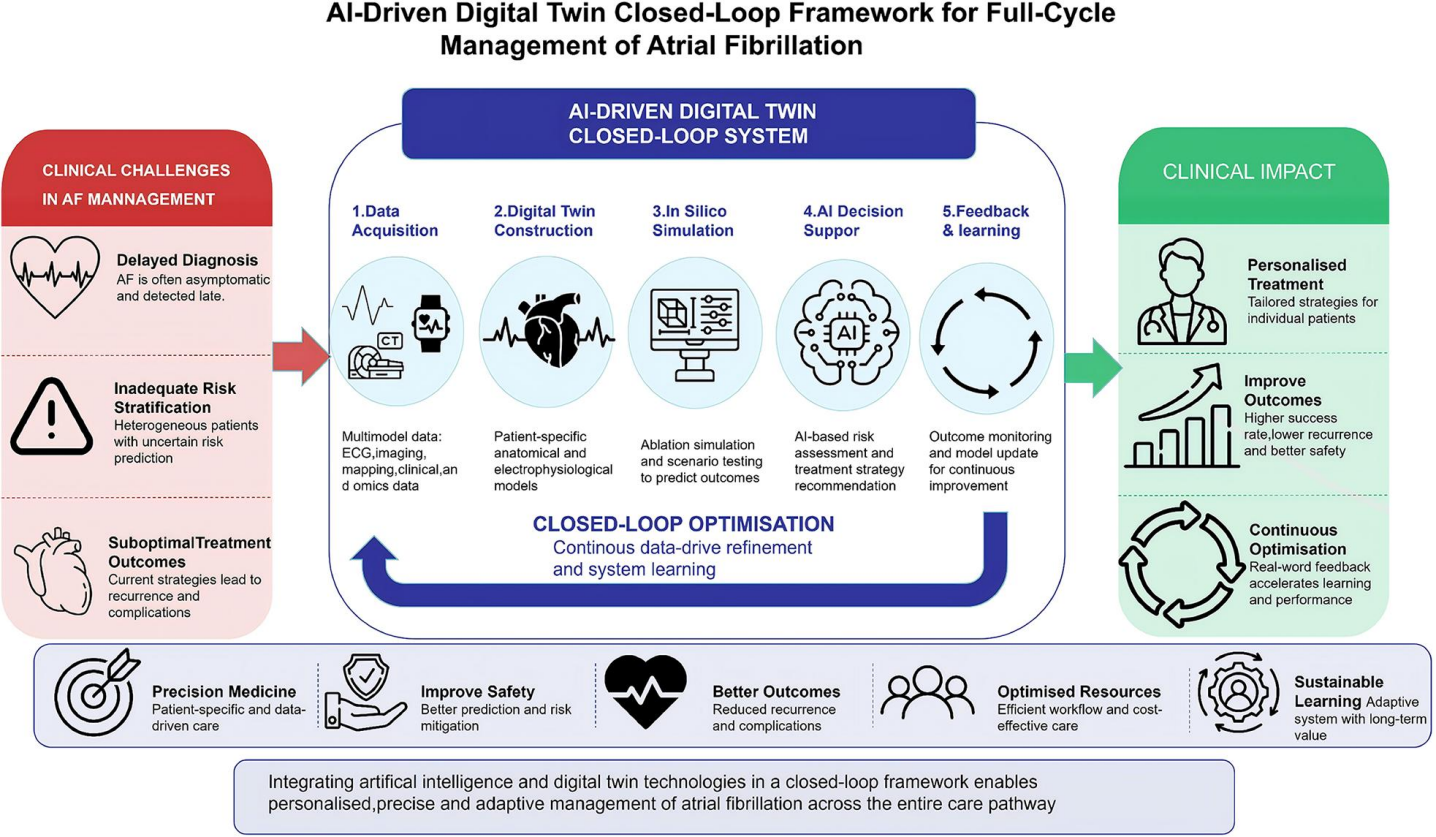
Graphical abstract: this figure summarises the AI-driven digital twin closed-loop framework for full-cycle management of atrial fibrillation (AF). (1)Left panel-Clinical challenges in AF management: Delayed diagnosis, inadequate risk stratification, and suboptimal treatment outcomes. These challenges are introduced in Section 1 (Introduction) and motivate the proposed framework. (2)Central closed-loop system -Five steps of the virtual closed-loop framework (Section 5.1): ① Data Acquisition (ECG, imaging, wearables, clinical data) ② Digital Twin Construction (patient-specific anatomy and electrophysiology) ③ In Silico Simulation (virtual testing of ablation, drugs, thrombus risk) ④ AI Decision Support (risk assessment, treatment strategy recommendation) ⑤ Feedback & Learning (real-world outcomes update the model). Right panel -Clinical impact: Personalised treatment, improved outcomes (higher success rate, lower recurrence), continuous optimisation through real-world feedback. Bottom statement summarises the paradigm shift from reactive, episodic care to prospective, continuous, personalised AF management. Readers are encouraged to refer to the main text for detailed evidence, technical validation, and discussion of limitations.

Before detailing the technical components, we provide a visual overview in the Graphical Abstract (Figure 2). The figure maps the five-step closed-loop system (data acquisition → digital twin construction → in silico simulation → AI decision support → feedback learning) to the clinical decision-making process described in Sections 5.1 and 5.2.

### 5.2 Evidence for application in key clinical scenarios

Virtual closed-loop frameworks have been preliminarily validated in several clinical scenarios. In the area of drug screening, Bai et al. built a digital twin platform that integrated *in vitro* measurements and machine learning, successfully achieving sex-specific identification of class III antiarrhythmic drugs (8). In the optimisation of ablation strategies, Jin et al. conducted simulation studies based on digital twins to evaluate the arrhythmogenic effects of pulmonary vein isolation gaps, thereby providing evidence for optimising procedural approaches (9). In the area of thrombus risk assessment, Qureshi et al. used MRI-based haemodynamic modelling to predict the risk of left atrial thrombus formation in patients with AF, introducing fluid dynamics simulations into the field of stroke prevention (10). Patient-specific models can also be used for pre-procedural planning of left atrial appendage occlusion, thereby improving device matching (33).

Taken together, these advances indicate that digital twin and artificial intelligence technologies are no longer purely conceptual; they have already been partially embedded into clinical workflows, particularly in early screening, intra-procedural guidance, and post-procedural risk stratification. For example, a patient preparing to undergo AF ablation can first undergo AI-assisted image segmentation to construct a personalised digital twin model, and then perform in silico testing of different ablation strategies; finally, the optimised plan can be used to guide intra-procedural decision-making. Importantly, the first prospective multicentre randomised controlled trial marks a key milestone, suggesting that this paradigm is moving from theoretical exploration towards evidence-driven clinical application.

### 5.3 Lessons from mature examples across other fields

Studies from other medical fields provide important references for virtual closed-loop management of AF. For example, Görtz et al. constructed digital twin models integrating multi-omics data in the field of uro-oncology to achieve personalised treatment predictions (14). Scibilia et al. demonstrated in the field of mathematical oncology that computational modelling is profoundly changing the clinical decision-making paradigm (34) and Younas et al. have also made progress in the field of tumour nanomedicine, reflecting a trend towards the convergence of smart targeting and personalised therapy (35). Recent reviews have also highlighted broader applications of Al in electrophysiology, including arrhythmia substrate characterization, automated electrogram interpretation, and procedural guidance systems (44).

These cross-disciplinary examples suggest that multimodal data integration, continuous model iteration, and embedding of clinical decision-making are key pathways for translating digital twins into clinical practice. Of note, the real-time dynamic nature of atrial electrophysiological processes imposes higher demands on model temporal resolution and computational efficiency.

## 6 Technology readiness assessment

Based on a comprehensive review of the existing literature, we qualitatively assessed the maturity and strength of evidence for each technology and constructed the following technology readiness–evidence strength matrix (Table 1), with the aim of providing researchers in the field with a reference roadmap for clinical translation.

**Table 1:** Technology readiness and evidence strength matrix.

Application	Technology readiness	Evidence strength	Representative references	Clinical readiness	Key performance metrics (sample size)
AI-assisted image segmentation	★★★★★	★★★★✰	(1, 17, 18)	Early commercial translation	Dice = 93.11% (*n* = 20 independent test)
Post-ablation recurrence prediction	★★★★✰	★★★✰✰	(4–6)	External validation needed	AUC 0.72–0.85 (*n* = 3,128,registry study)
Intra-operative real-time 3D reconstruction	★★★★✰	★★★✰✰	(7, 26)	Partially commercialised	Reconstruction time ≈ 65 s (*n* = 28)
Inverse solving of electrophysiological parameters	★★★✰✰	★★★✰✰	(2, 20–22)	Research stage	*r* ≈ 0.96 for activation time (*n* = 9 patients)
Digital twin-based ablation simulation	★★✰✰✰	★★✰✰✰	(9)	Early exploration	Simulation results in silico (*n* = 50)
In silico drug screening	★★✰✰✰	★✰✰✰✰	(8)	Proof of concept	Sex-specific identification (*n* = 201 patients)
Thrombosis risk assessment	★★✰✰✰	★★✰✰✰	(10)	Research stage	Haemodynamic model validation (*n* = 9)
Pre-procedural planning for LAA closure	★★★✰✰	★★✰✰✰	(33)	Exploratory study	Device matching simulation (*n* = 4)

Data summarized from references: for AI-assisted image segmentation (1, 17, 18), for post-ablation recurrence prediction (4-6), for intra-operative real-time 3D reconstruction (7, 26), for inverse solving of electrophysiological parameters (2, 20-22), for digital twin-based ablation simulation (9), for in silico drug screening (8), for thrombosis risk assessment (10), for preprocedural planning for LAA closure (33).

Interpretation of the matrix: applications with a relatively high level of technology readiness are currently concentrated in the fields of image processing and risk prediction, some of which have already entered the early commerical translation. The technology readiness of electrophysiological modelling and in silico testing remains at a moderate level, requiring further clinical validation. The overall closed-loop system integrating multimodal data is still in the early exploration stage, representing a key development direction for the next 3–5 years.

## 7 Discussion: key issues and future directions in paradigm shift

### 7.1 What are the limitations of current research?

Although digital twin and artificial intelligence are developing rapidly in the field of AF, current research still has the following shortcomings.

1. External validation is generally lacking. Most models remain at the level of single-centre, retrospective data and lack multicentre, prospective validation. Although a few studies have performed external validation (36), their overall number is still limited, and the generalisability of the models falls short of what is needed to meet clinical demands.
2. Multimodal integration is still at a “patchwork” stage. Most existing studies remain at the level of simple data-level integration and have not yet achieved true deep fusion. Some studies have demonstrated that multimodal fusion can improve predictive performance (AUC increased from 0.79 to 0.85) (29); however, the relevant evidence remains relatively limited.
3. There is a gap in the evidence chain for clinical outcomes. Although the randomized controlled trial by Deisenhofer et al. provided the first prospective evidence supporting Al-guided AF management, additional multicenter studies are still needed to determine its impact on long-term clinical endpoints (11).
4. Issues related to clinical implementation and human–AI collaboration remain inadequately addressed. Brunyé et al., from a human–computer interaction perspective, pointed out that the clinical adoption rate of AI-assisted diagnostic systems often falls short of what would be expected from their technical performance, and a key reason is that smooth integration of algorithmic output into clinical decision-making processes has not been adequately considered (37). Likewise, Zhang et al. found that when AI is involved in decision-making, patients’ moral judgments of physicians exhibit a “diffusion of responsibility” phenomenon (38), suggesting that non-technical factors may become hidden barriers to clinical translation.

### 7.2 Distinctive contributions and clinical perspective of the present review

Unlike previous reviews that have mostly focused on a single technology (15, 16), the present review starts from the clinical pathway and integrates the applications of digital twins and artificial intelligence within a unified clinical workflow framework. The main contributions are reflected in the following three aspects.

1. It provides the first systematic elucidation of the virtual closed-loop management framework comprising “data sensing → model construction → in silico testing → clinical decision → feedback optimisation”, offering a holistic perspective on full-cycle AF management.
2. It constructs a technology readiness–evidence strength matrix, providing a stratified assessment of the level of clinical translation for different technologies and helping to identify research priorities.
3. With a focus on clinical application, it proposes key research directions for the next 3–5 years, providing a roadmap for transitioning from technical exploration to clinical translation in this field.

### 7.3 Priorities for future research

Based on the limitations of current research, we propose the following priority directions for future research.

Priority A: Multicentre prospective validation studies. The most urgent task is to perform multicentre, prospective validation of the predictive models and digital twin construction methods that have already been developed. It is recommended to adopt an iterative “development–validation–update” paradigm, evaluate the generalisability of the model in new cohorts (36), and perform calibration updates tailored to population differences. In the next 2–3 years, at least 3–5 models that have undergone external validation should enter the stage of clinical pilot testing.
Priority B: Ablation randomized controlled trial (RCT) driven by artificial intelligence and digital twins. The RCT conducted by Deisenhofer et al. represents a pioneering study in this field (11). Over the next 5 years, well-designed randomised controlled trials are needed to evaluate their impact on key endpoints such as recurrence rate, procedure duration, and complications, and to demonstrate the clinical superiority of virtual closed-loop management over conventional care, with a sample size of approximately 400–600 patients and a follow-up duration of no less than 12 months.
Priority C: Investigating model explainability and clinical trust mechanisms. The five human–computer interaction questions proposed by Brunyé et al. warrant in-depth investigation (37): How should the visualisation formats of AI outputs be designed to align with clinicians’ cognitive habits? How can “algorithmic confidence” be quantified and effectively communicated to decision-makers? The study by Zhang et al. showed that patient acceptance of AI involvement in decision-making needs to be systematically assessed (38). Research in this area will help remove psychological barriers to clinical acceptance.
Priority D: Development of multimodal deep fusion algorithms. The current “patchwork” approach to integration has major limitations and fails to make full use of the complementary information across different modalities. Future efforts should focus on developing novel fusion algorithms based on graph neural networks and attention mechanisms to enable collaborative modelling of imaging, electrophysiological, and genomics data. The study by Tang et al. demonstrated the feasibility of this direction (29). However, further expansion is still needed in terms of technical depth and breadth of application.
Priority E: Validation of closed-loop systems using real-world data. Deploy small-scale closed-loop systems in real-world clinical settings (e.g., an “AI-assisted image segmentation and ablation planning” module) and prospectively collect process metrics (e.g., usage rate, rate of decision change) and outcome-related indicators to validate their feasibility and added value in real-world workflows. This type of “effectiveness research” will help bridge the gap between randomised controlled trials (RCTs) and real-world practice.

## 8 Challenges, ethics, and pathways to clinical integration

### 8.1 Technical challenges

Although digital twins and artificial intelligence have shown considerable promise in AF management, their clinical translation still faces multifaceted challenges. First, data standardisation and interoperability remain unresolved. The formats of imaging and electrophysiological data vary considerably across different devices and centres, which limits multicentre data integration and model generalisability (39, 40). Second, insufficient model explainability remains a major factor limiting clinical application. Although some progress has been made with explainable machine learning methods (5), their application in complex mechanistic models still needs further development. Furthermore, the balance between computational efficiency and real-time performance still requires optimisation. Although current studies have made progress in improving model computational efficiency (21, 41), a considerable gap remains in meeting the demands of real-time clinical decision-making. Finally, external validation using multicentre, large-scale cohorts remains insufficient, and the generalisability of models needs to be further improved (36).

### 8.2 Pathways for integration with existing clinical practice

From technical validation to routine clinical use, the integration pathway can be advanced in phases: Phase I (short term, approximately 1–3 years): Embedding decision-support tools. Integrate AI-assisted image segmentation and risk prediction models into existing electrophysiological recording systems (e.g., CARTO, EnSite) to serve as “decision-support tools” to aid clinical judgment, rather than replacing physician decision-making. At present, preliminary applications of relevant research have been achieved in intra-operative three-dimensional reconstruction techniques and pulmonary vein isolation identification algorithms (7, 26).

Phase II (mid-term, approximately 3–5 years): Building multicentre data platforms. Establish regional multicentre data-sharing platforms to promote validation and optimisation of models across different populations (4, 30). At the same time, the hardware infrastructure should be improved. This includes the configuration of computational resources in electrophysiology catheterisation laboratories (e.g., edge computing servers) and the standardisation of interfaces for hospital information system (HIS).

Phase III (long-term, approximately 5–10 years): Standardised application and incorporation into guidelines. Supported by high-quality clinical evidence (11), virtual closed-loop management can be incorporated into clinical guidelines, gradually transitioning from a “decision-support tool” to a “standardised diagnostic and treatment workflow”, and a system for quality control and adverse event reporting should be established.

### 8.3 Ethical, regulatory, and social considerations

The application of artificial intelligence and digital twins in healthcare raises ethical and regulatory concerns. Brunyé et al. emphasised that effective integration of algorithmic output and clinical judgment is key in AI-assisted diagnosis (37). From a sociological perspective, Gross et al. pointed out that issues such as data justice and capital flows raised by AI in healthcare are gradually gaining attention (42). The study by Zhang et al. explored the potential “diffusion of responsibility” effect when AI is involved in decision-making, which may influence patient acceptance of AI recommendations (38). The systematic review by Gao et al. provided a comprehensive overview of the technical efficacy, ethical dilemmas, and governance pathways of AI applications in the field of public health (43). From a regulatory perspective, digital twin and AI systems need to have their status as “medical devices” clearly defined, and a complete evaluation pathway from algorithmic validation to clinical access should be established to ensure their safety and effectiveness. Beyond technical performance, practical implementation barriers should also be considered.AI-assisted digital twin systems may introduce automation bias, where clinicians over-rely on algorithmic recommendations despite uncertain model confidence. Additional concerns include cybersecurity risks, medico-legal liability, interoperability with existing electrophysiological platforms, data ownership, and increased workflow burden associated with acquisition of high-quality imaging and electrophysiological datasets. Future studies should therefore evaluate not only predictive accuracy, but also cost-effectiveness, workflow feasibility, and clinician acceptance in real-world environments.

### 8.4 Clinical translation and AI governance

The clinical translation of AI-assisted AF management systems should follow emerging standards for trustworthy artificial intelligence in healthcare. CONSORT-AI and SPIRIT-AI frameworks emphasize transparent reporting, external validation, calibration assessment, dataset shift monitoring, and continuous algorithm updating. In AF management, model performance may vary across institutions because of differences in imaging protocols, electrophysiological mapping systems, patient populations, and workflow structures. Therefore, future AI-enabled digital twin systems should incorporate explainability mechanisms, continual recalibration strategies, and version control frameworks to ensure reproducibility and clinical reliability.

Despite promising preliminary findings, most currently available AI-assisted digital twin systems remain at the proof-of-concept or early translational stage. Further efforts are needed to evaluate long-term clinical benefit, workflow feasibility, cost-effectiveness, and physician acceptance before widespread clinical adoption can be achieved.

### 8.5 Predictions for development pathways over the next 3–5 years

Based on current technological advances and the progress of clinical trials, the virtual closed-loop management of AF is expected to gradually evolve in the following ways:

2026–2027: Systems integrating AI-based image segmentation and electrophysiological modelling are expected to undergo preliminary clinical application in pre-procedural planning (1, 7, 17).
2027–2028: Based on the technical foundation provided by existing predictive models and mechanistic simulation methods, multicentre prospective studies will be gradually carried out to further validate their clinical value in optimising ablation strategies (4, 30).
2028–2029: Supported by evidence from randomised controlled trials (11), relevant technologies may enter clinical recommendation pathways.
Around 2030: Virtual closed-loop management is expected to achieve standardised application in specific clinical scenarios (21, 41).

## 9 Conclusion

The deep integration of digital twin and artificial intelligence provides a novel technical pathway for full-cycle management of atrial fibrillation. By integrating multimodal imaging, electrophysiological, and clinical data into patient-specific models, this paradigm enables full-cycle decision support spanning early warning, treatment planning, procedural guidance and follow-up management. Existing studies have demonstrated potential clinical value in areas such as image segmentation, recurrence prediction, and optimisation of ablation strategies.

However, the field as a whole is still at a developmental stage. Issues such as data standardisation, multicentre validation, and model explainability have not yet been fully resolved, and the impact on clinical hard endpoints such as stroke and death still lacks sufficient supporting evidence. Therefore, at the current stage, these technologies should be positioned as decision-support tools to aid clinical decision-making, rather than replacing traditional diagnostic and treatment paradigms.

With further optimisation of algorithms, improvements in computational power, and the conduct of high-quality clinical studies, digital twins and artificial intelligence are expected to be gradually integrated into the AF management workflow. In specific clinical scenarios, this model may improve diagnostic and treatment efficiency and facilitate personalised decision-making. In the future, further multicentre studies and randomised controlled trials are needed to validate its clinical value and promote its standardised application.
